# Comparative genotypic analysis of Candida krusei and Candida parapsilosis isolates from human and animal clinical samples through MLVA and RAPD-PCR profiling

**DOI:** 10.3205/dgkh000653

**Published:** 2026-06-05

**Authors:** Armin Rezaie Dorostkar, Mansour Bayat, Kumars Amini, Mohammad Hossein Yadegari

**Affiliations:** 1 Department of Veterinary Pathobiology, SR.C., Islamic Azad University, Tehran, Iran; 2 Department of Microbiology, Sav.C., Islamic Azad University, Saveh, Iran; 3 Department of Medical Mycology, Faculty of Medical Sciences, Tarbiat Modares University, Tehran, Iran

**Keywords:** Candida krusei, Candida parapsilosis, DNA fingerprinting, molecular typing, genetic variation

## Abstract

**Introduction:**

*Candida (C.) parapsilosis* and *C. krusei* are significant fungal pathogens in both human and animal clinical settings. Molecular typing techniques, such as Multiple-Locus Variable Number Tandem Repeat Analysis (MLVA) and Random Amplified Polymorphic DNA Polymerase Chain Reaction (RAPD-PCR), play a crucial role in understanding the genetic diversity and epidemiology of these pathogens. The aim of the present study is to genotype the strains of *C. krusei* and *C. parapsilosis* isolated from human and animal clinical samples using MLVA and RAPD-PCR methods.

**Methods:**

A total of 30 human clinical samples, including 15 *C. krusei* and 15 *C. parapsilosis* candidates, were obtained from Imam Khomeini Hospital, and 30 animal samples, including 15 Candida krusei and 15 *C. parapsilosis*, were collected from veterinarians in dairy farms around Tehran. The study aimed to genotype strains of *C. krusei* and *C. parapsilosis* using MLVA and RAPD-PCR methods. Phenotypic confirmation of the Candida species was conducted, and genes related to virulence and antifungal resistance were examined using Multiplex-PCR. The genetic diversity of the strains was assessed using MLVA and RAPD-PCR methods, and dendrograms were constructed for each species.

**Results:**

The samples received from dairy farms or collected from the hospital were confirmed using phenotypic methods. The frequency of genes CDR1, CDR2, and MDR1 in *C. krusei* human samples were 66/86%, 33/13%, and 0%, respectively; in candidate *C. krusei* animal samples, they were 66/86%, 66/46%, and 20%; in *C. parapsilosis* human samples 80%, 40%, and 40%; in *C. parapsilosis* animal samples 66/86%, 66/66%, and 40%. Genetic relatedness analysis of 30 *C. krusei* samples was performed using microsatellite typing with the PHYLOViZ, which showed four clusters and four keys in the phylogenetic tree using the UPGMA method. Similarly, the genetic relatedness of 30 candidate *C. parapsilosis* samples was analyzed, which showed six clusters and six keys in the phylogenetic tree using the UPGMA method. Based on the RAPD-PCR results, all *C. krusei* samples were divided into six groups at a similarity level of 59%, with a simplicity index of 31/0. Similarly, all *C. parapsilosis* samples were divided into four groups at a similarity level of 59%, with a simplicity index of 35/0.

**Conclusion:**

The RAPD-PCR method has shown limited discriminatory power compared to MLVA for genetic diversity assessment. However, the combination of MLVA and RAPD-PCR techniques provides a comprehensive approach to molecular typing and genetic characterization of *C. parapsilosis* and *C. krusei* isolates. This integrated approach offers valuable insights into the genetic diversity, epidemiology, and potential transmission dynamics of these strains, facilitating the development of targeted diagnostic and therapeutic strategies.

## Zusammenfassung

**Einleitung:**

*Candida (C.) parapsilosis* und *C. krusei* sind bedeutende Pathogene in der klinischen Umgebung von Mensch und Tier. Molekulare Typisierungsverfahren wie die MLVA-Analyse (Multiple-Locus Variable Number Tandem Repeat Analysis) und die RAPD-PCR (Random Amplified Polymorphic DNA Polymerase Chain Reaction) spielen eine entscheidende Rolle für das Verständnis der genetischen Vielfalt und Epidemiologie dieser Krankheitserreger. Ziel der vorliegenden Studie ist es, die aus klinischen Proben von Mensch und Tier isolierten Stämme von *C. krusei* und *C. parapsilosis* mittels MLVA- und RAPD-PCR-Methoden zu genotypisieren.

**Methode:**

30 klinische Proben von Patienten, darunter 15 *C. krusei*- und 15 *C. parapsilosis*-Kandidaten, wurden aus dem Imam-Khomeini-Krankenhaus entnommen, und 30 Tierproben, darunter 15 C. krusei und 15 *C. parapsilosis*, wurden von Tierärzten in Milchviehbetrieben in der Umgebung von Teheran gesammelt. Ziel war die Genotypisierung mittels MLVA- und RAPD-PCR-Verfahren. Die Candida-Spezies wurden phänotypisch bestätigt, und Gene, die mit der Virulenz und der antimykotischen Resistenz zusammenhängen, wurden mit Multiplex-PCR untersucht. Die genetische Vielfalt der Stämme wurde mit MLVA- und RAPD-PCR-Methoden bewertet, und für jede Art wurden Dendrogramme erstellt.

**Ergebnisse:**

Die Proben wurden mittels phänotypischer Methoden bestätigt. Die Häufigkeit der Gene CDR1, CDR2 und MDR1 in den *C. krusei*-Probe, bei den *C. parapsilosis*-Proben 80%, 40% bzw. 40%, bei den *C. parapsilosis*-Tierproben 66,9%, 66,7% bzw. 40%. Die genetische Verwandtschaftsanalyse der 30 *C. krusei*-Proben mittels Mikrosatelliten-Typisierung mit PHYLOViZ ergab vier Cluster und vier Schlüssel im phylogenetischen Baum mittels UPGMA-Methode. Die Analyse der genetischen Verwandtschaft der 30 *C. parapsilosis*-Proben ergab sechs Cluster und sechs Schlüssel im phylogenetischen Baum. Basierend auf den RAPD-PCR-Ergebnissen wurden alle *C. krusei*-Proben bei einem Ähnlichkeitsniveau von 59% in sechs Gruppen mit einem Einfachheitsindex von 31,0 unterteilt. Ebenso wurden alle *C. parapsilosis*-Proben bei einem Ähnlichkeitsniveau von 59% in vier Gruppen mit einem Einfachheitsindex von 35,0 unterteilt.

**Schlussfolgerung:**

Die RAPD-PCR-Methode zeigte im Vergleich zur MLVA eine begrenzte Diskriminierungskraft bei der Bewertung der genetischen Vielfalt. Die Kombination der MLVA- und RAPD-PCR-Techniken bietet jedoch einen umfassenden Ansatz zur molekularen Typisierung und genetischen Charakterisierung von *C. parapsilosis*- und *C. krusei*-Isolaten. Dieser integrierte Ansatz liefert wertvolle Einblicke in die genetische Vielfalt, Epidemiologie und potenzielle Übertragungsdynamik dieser Stämme und erleichtert die Entwicklung gezielter diagnostischer und therapeutischer Strategien.

## Introduction

The species of *Candida (C.)* commonly associated with human and animal diseases clinically include *C. albicans*, *C. tropicalis*, *C. glabrata*, *C. parapsilosis*, and *C.* krusei. *C. parapsilosis* and *C. krusei* are two significant fungal pathogens that can cause a range of infections in both humans and animals. *C. parapsilosis* is a ubiquitous organism, and has even been isolated from soil. It is considered the most common non-*C. albicans* *candidiasis/* infection and typically requires a host with an impaired immune system [1]. *C. parapsilosis* is associated with conditions such as endocarditis, meningitis, septicemia, peritonitis, arthritis, endophthalmitis, keratitis, otitis, cystitis, and skin infections in humans. In comparison to other medically important *Candida* species, *C. krusei* has been isolated from a wide variety of natural habitats such as barley, fruits, sewage, silage, soil, and food (including dairy and meat products, pickles, sugar, and syrup). As a result, it is widely distributed in nature and is considered an opportunistic saprophyte [2], [3]. It is also found in poultry and marine birds. Generally, *C. krusei* is considered a transient colonizer in humans and is rarely isolated from mucosal surfaces of different patient groups, being more commonly found as a commensal on the mucosal surfaces of healthy individuals [4].

From a genetic standpoint, genetic diversity plays a crucial role in enabling species or populations to endure a broad spectrum of environmental changes, encompassing bacteria, fungi, and beyond. Furthermore, genetic diversity serves as a valuable mechanism for safeguarding evolutionary potential. Generally, heightened genetic diversity empowers an organism to more effectively adapt to novel selective pressures. Given selective pressure exerted by external antifungal agents, greater genetic diversity within fungal populations increases their chances of survival. In essence, antifungal agents act as selective forces within the framework of Darwinian evolution [5]. In instances where the genetic diversity of fungal populations is substantial, certain individual fungi may succumb to the pressures of drug selection, while others with different genetic traits may survive. Consequently, it is plausible to posit that microbial populations exhibiting greater genetic diversity are predisposed to the development of antimicrobial drug resistance [6]. Nevertheless, to the best of our knowledge, no research has been undertaken to date with the specific objective of characterizing the population-level genetic traits of *C. krusei* and *C. parapsilosis*..

Molecular typing techniques, such as Multiple-Locus Variable Number Tandem Repeat Analysis (MLVA) and Random Amplified Polymorphic DNA Polymerase Chain Reaction (RAPD-PCR ), are essential tools for understanding the genetic diversity and epidemiology of these pathogens [7].

MLVA is a powerful tool for analyzing the genetic variability of tandem repeat regions in the genome, providing high-resolution differentiation of strains [8]. Similarly, RAPD-PCR is a useful method for generating unique DNA fingerprints for each isolate, enabling the identification of distinct genetic patterns within the isolates [9].

In the context of *C. parapsilosis* and *C. krusei*, MLVA and RAPD-PCR can provide valuable insights into the genetic diversity, epidemiology, and potential transmission dynamics of these fungal pathogens. For instance, a study using RFLP typing revealed indistinguishable restriction patterns between *C. krusei* strains isolated from each patient and their respective environment, suggesting an endogenous origin of infectious episodes [10]. Another study using BssHII and EagI [11] RFLP followed by PFGE analysis traced *C. parapsilosis* prosthetic-valve endocarditis to the contaminated hands of healthcare workers, highlighting the importance of targeted infection control measures.

The aim of this study was to investigate the genetic diversity and epidemiology of *C. parapsilosis* and *C. krusei* isolates obtained from clinical samples from both animals and humans. Specifically, the study employed MLVA to assess the genetic variability based on tandem repeat regions in the genome, allowing high-resolution differentiation of strains. Additionally, the study intended to use RAPD-PCR to generate unique DNA fingerprints for each isolate, enabling the identification of distinct genetic patterns within the isolates. By applying these molecular typing techniques, the study sought to provide insights into the genetic diversity, epidemiology, and potential transmission dynamics of *C. parapsilosis* and *C. krusei*, ultimately contributing to the development of targeted diagnostic and therapeutic strategies for the effective management of infections caused by these species.

## Materials and methods

### Origin of samples, cultivation and phenotypic identification

A total of 30 clinical samples, comprising 15 *C. k*rusei and 15 C. parapsilosis samples, were obtained from the Imam Khomeini Hospital laboratory. Additionally, 30 animal samples, including 15 *C. krusei* and 15 *C. parapsilosis* samples, were received from veterinarians in dairy farms around Tehran. All the collected samples were inoculated onto Sabouraud dextrose agar and incubated. After the colonies grew, a loop was used to transfer some of them to chromogenic agar, where they were incubated at 35°C for 48 hours. The color of the colonies grown on chromogenic agar was examined for the identification of the isolated strains. To perform this test, colonies grown in Sabouraud dextrose agar that had been incubated for 24 to 48 hours and fresh serum were used. A small amount of the yeast colonies were taken with an inoculating loop and suspended in fresh serum to prepare a suspension. The suspension was kept at 37°C for 2 to 3 hours. A drop of the suspension was taken with an inoculating loop and placed on a sterile slide, then covered with a coverslip. The presence or absence of hyphae (short and early hyphae directly produced from the swollen cells) was examined using a bright-field microscope.

### Genomic DNA extraction

Genomic DNA extraction was carried out as follows: The *Candida* strains were cultured on Sabouraud Dextrose Agar (SDA; Difco) for 48 hours at 28°C to establish a pre-inoculum. Subsequently, 3 x 10^8^ CFU/ml were inoculated into 50 ml of SDA and incubated for an additional 48 hours at 28°C. The resulting cellular biomasses were harvested by centrifugation at 10,000 x g, followed by resuspension in 5 ml of 0.1 M sodium citrate/1.1 M sorbitol buffer (pH 5.5) supplemented with 5 mg/ml of glucanase enzyme. This suspension was then subjected to a 3-hour incubation at 33°C in a shaking water bath to generate protoplasts. The protoplasts obtained were then transferred to 5 ml of lysing buffer (composed of 0.04 M Tris HCl, pH 8.0; 0.20 M NaCl, SDS, and 0.01 M Na2 EDTA), washed thrice with 5 ml of phenol-chloroform, and subsequently precipitated using absolute ethanol and 0.3M NaCl. The resulting precipitate was centrifuged, washed twice with 70% ethanol, dried, and finally resuspended in 100 ml of 0.10 mM Tris HCl (pH 7.5). DNA aliquots were then diluted to a concentration of 50 ng/ml in preparation for the RAPD reaction [12].

### Multiplex PCR

The DNA extracted from the samples was used for molecular identification of antifungal resistance genes. Multiplex PCR was utilized for simultaneous identification of genes, aiming for time and cost efficiency. The primers [13], contents, and reaction programs used are presented in Table 1 (Tab. 1).

### RAPD-PCR

RAPD profiles were generated using a 30-µl reaction volume comprising a 1X buffer (Promega), 0.2 mM each of dATP, dGTP, dCTP, and dTTP (Promega), 50 ng of genomic DNA, 2 mM of MgCl2 (Promega), 160 nM of primer (Operon), and 1 unit of Taq thermostable DNA polymerase (Promega). The amplification protocol entailed 35 cycles, with denaturation at 95°C for 45 seconds, primer annealing at 36°C for 2 minutes, and extension at 72°C for 2 minutes. Initial denaturation lasted 5 minutes in the first cycle, while the final extension was set to 7 minutes in the last cycle.

Decamer primers from the OPERON kit (OPA 01, 02, 03, 07, 08, 09, and 10) along with arbitrary primers SOY, RP1-4, RP-2, and RP4-28 were utilized for the reactions. Additionally, amplification was conducted using ribosomal primers NS1, NS2, ITS1, and ITS-424, with the melting temperature adjusted to 45°C. The specific primer sequences [14] can be found in Table 2 (Tab. 2).

Fingerprints were generated through electrophoresis of 10-µl aliquots of the reaction on 1.5% agarose gels, which were electrophoresed in TBE (0.45 M Tris borate, 0.001 M EDTA) buffer at 120 V for 90 minutes. Subsequently, the gels were stained with 1 µg/ml ethidium bromide and visualized under UV light using a Polaroid camera (Model DS-34) equipped with black and white film (Type 667, Polaroid Corp.). In each experimental run, the base pair sizes were determined by referencing size markers present in every gel, such as DNA lambda/Hind III or 100-bp ladder from Gibco-BRL.

### Multiple-locus variable-number tandem-repeat analysis (MLVA)

For each primers (Table 3 (Tab. 3)) set [8], polymerase chain reactions (PCRs) were carried out in a final volume of 20 µl, comprising 1 µl of DNA, deoxynucleoside triphosphates at a concentration of 200 µM each, a forward primer, a 5'-dye-labeled reverse primer at 0.25 µM each, and 1 unit of Taq DNA polymerase (Promega, Madison, WI). The thermal cycling conditions consisted of an initial denaturation step at 95°C for 5 minutes, followed by 35 cycles of denaturation at 95°C for 30 seconds, annealing at 52°C for 30 seconds, extension at 72°C for 45 seconds, and a final extension step at 72°C for 10 minutes. Subsequently, amplicons from each PCR reaction for a given isolate were combined prior to multiplex fragment analysis using a Ceq 8000 Genetic Analyzer (Beckman Coulter, Fullerton, CA, USA). Strain IHEM 9670 served as a control in all experiments. To facilitate interlaboratory comparisons of multiple-locus variable-number tandem repeat analysis (MLVA) results, the amplicon sizes were reported as the exact sequence length (determined through sequencing of representative alleles at each locus), given the dependency of allele sizing on dyes and the specific analyzer utilized for electrophoresis. The method's reproducibility and stability were evaluated following established protocols. Primer specificity was verified by examining 11 non-*C. glabrata* reference strains, including *C. albicans* IHEM 9559, *C. dubliniensis* IHEM 14280, *C. tropicalis* CBS 1920, *C. parapsilosis* IHEM 9557, and *C. krusei* IHEM 9560.

The discriminatory power (D) of Multiple Loci VNTR Analysis (MLVA) was determined using the formula proposed by Hunter and Gaston [15]. To classify the genetically unrelated isolates based on their distance, hierarchical clustering analysis was conducted using R software along with the pvclust package. The potential associations between genotypes and the origins of isolates (clinical data, sex, ward, and anatomical sites) were examined through hierarchical clustering analysis combined with canonical discriminant analysis using Tanagra software.

### Dendrogram plot

The dendrogram construction and analysis of bands obtained from electrophoresis of the study samples were performed using NTSYS version 2.02e software. Initially, scoring of the bands resulting from the marker electrophoresis was done as quantitative data of zero (0) and one (1) (presence or absence of a band). Subsequently, genetic similarity based on the zero and one data was calculated using Jaccard and Dice coefficients and simple matching. To assess the efficiency of the RAPD-PCR method through cluster analysis based on similarity coefficients, the coefficient of cophenetic correlation was utilized. For strain grouping, cluster analysis using the UPGMA method based on the similarity coefficient with the highest cophenetic correlation coefficient was employed.

## Results

**Figure 1:**
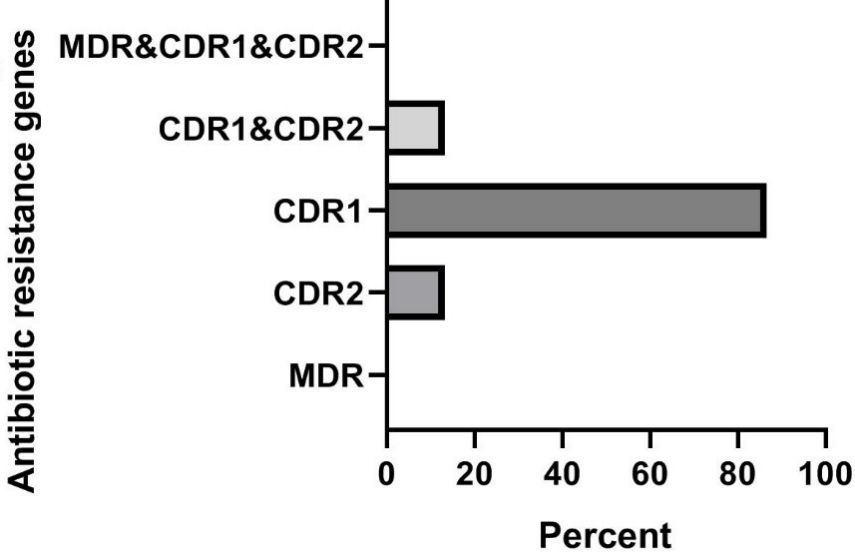
Investigation of the frequency of antibiotic resistance genes in clinical samples of *C. krusei*

**Figure 2:**
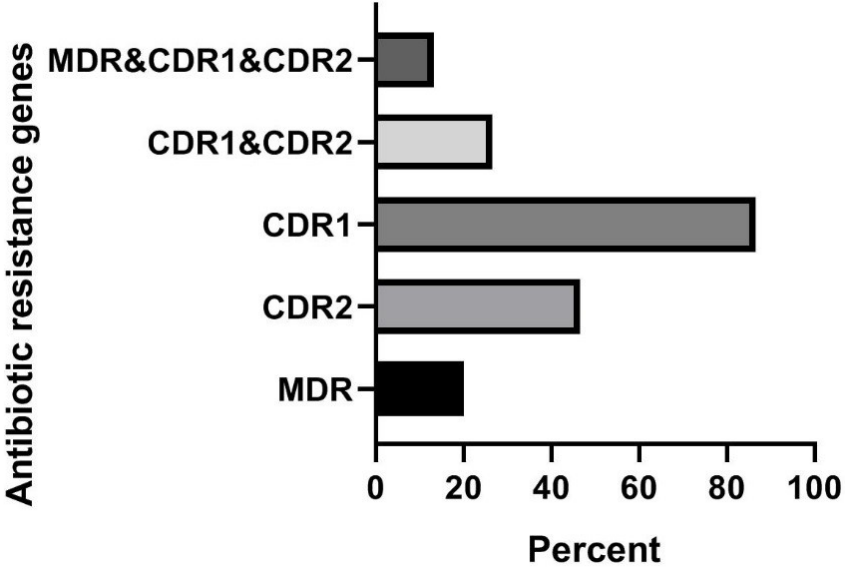
Investigation of the frequency of antibiotic resistance genes in animal samples of *C. krusei*

Based on the results presented in Figures 1 (Fig. 1), in clinical isolates of *C. krusei*, the frequencies of the genes were as follows: MDR1 gene 0% (0 out of 15 samples), CDR2 gene 13.3% (2 out of 15 samples), CDR1 gene 86.7% (13 out of 15 samples), the simultaneous presence of the CDR1 and CDR2 genes 13.3% (2 out of 15 samples), and the frequency of the simultaneous presence of the three genes MDR1, CDR2, and CDR1 was also 0% (0 out of 15 samples). In animal isolates of *C. krusei*, the frequencies of the genes were: MDR1 gene 20% (3 out of 15 samples), CDR2 gene 46.7% (7 out of 15 samples), CDR1 gene 86.7% (13 out of 15 samples), simultaneous presence of the CDR1 and CDR2 genes 26.7% (4 out of 15 samples), and the frequency of the simultaneous presence of the three genes MDR1, CDR2, and CDR1 was also 13.3% (2 out of 15 samples) (Figure 2 (Fig. 2)).

In clinical isolates of *C.* *parapsilosis*, the frequencies of the genes were: MDR1 gene 40% (6 out of 15 samples), CDR2 gene 40% (6 out of 15 samples), CDR1 gene 80% (12 out of 15 samples), simultaneous presence of CDR1 and CDR2 genes 6.7% (1 out of 15 samples), and the frequency of the simultaneous presence of the three genes MDR1, CDR1, and CDR2 was also 33.3% (5 out of 15 samples) [1], [2]. In animal isolates of *C. parapsilosis*, the frequencies of the genes were: MDR1 gene 40% (6 out of 15 samples), CDR2 gene 66.7% (10 out of 15 samples), CDR1 gene 86.7% (13 out of 15 samples), simultaneous presence of the CDR1 and CDR2 genes 33.3% (5 out of 15 samples), and the frequency of the simultaneous presence of the three genes MDR1, CDR1, and CDR2 was also 40% (6 out of 15 samples).

**Figure 3:**
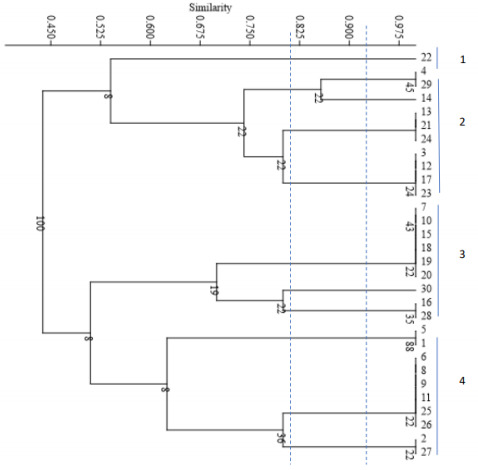
Phylogenetic tree based on the analysis of microsatellite results of *C. krusei* samples

**Figure 4:**
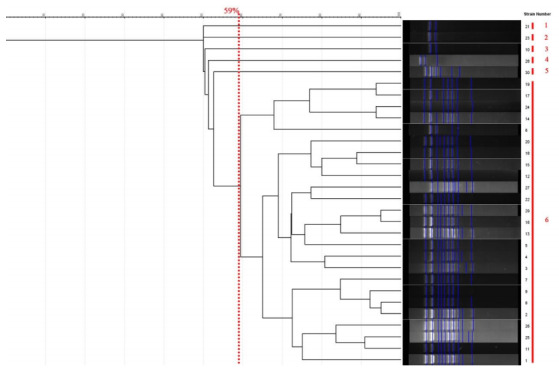
The dendrogram is drawn based on RAPD-PCR for *C. krusei*

All strains of *C. krusei* examined at a similarity level of 59% were divided into 6 distinct groups, such that 1 isolate was placed in the first, second, third, fourth, and fifth groups, and 25 isolates were placed in the sixth group. The results of this part of the study are presented Figure 3 (Fig. 3). The Simpson's coefficient calculated for RAPD-PCR was 31.0, indicating the low discriminatory power of this method for genotyping and polymorphism analysis of *C. krusei* (Figure 4 (Fig. 4))

**Figure 5:**
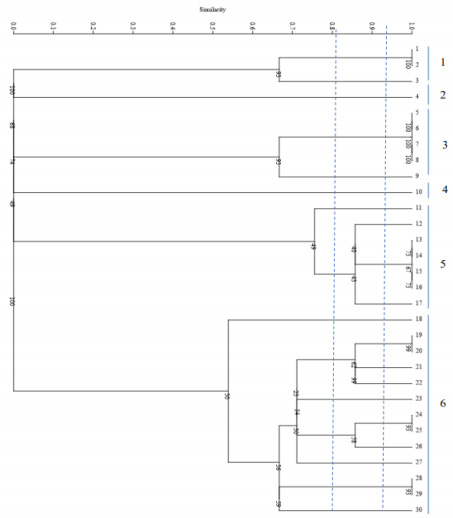
Phylogenetic tree based on the analysis of microsatellite results of *C. parapsilosis* samples

**Figure 6:**
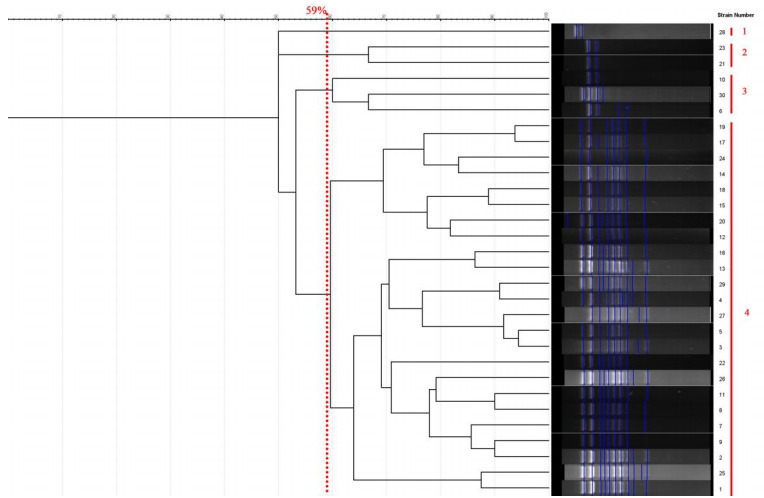
Dendrogram based on RAPD-PCR for *C. parapsilosis*

All strains of *C. parapsilosis* examined at a similarity level of 59% were divided into 4 distinct groups, such that 1 isolate was placed in the first group, 2 isolates in the second group, 3 isolates in the third group, and 24 isolates in the fourth group. The results of this part of the study are presented in Figure 5 (Fig. 5). The Simpson's coefficient calculated for RAPD-PCR was 35.0, indicating the low discriminatory power of this method for genotyping and polymorphism analysis of *C. parapsilosis* (Figure 6 (Fig. 6)).

## Discussion

*Candida* species have emerged as significant contributors to nosocomial infections, particularly in intensive care units, highlighting the issue of cross-transmission [16]. C. parapsilosis has emerged as a common cause of bloodstream infections (BSI), which, along with other invasive forms of candidiasis (e.g., non-albicans forms), is the most common hospital-acquired systemic fungal infection [17]. Although the prevalence of non-albicans *Candida* species may vary significantly depending on geographic location, *C. parapsilosis* is the second or third most commonly isolated *Candida* species (after *C. albicans*) from hospitalized patients worldwide [18].

The evolving challenges posed by *Candida* infections necessitate standardized approaches for characterizing strains to pinpoint hospital clusters. The increasing morbidity and mortality associated with candidiasis, caused by pathogenic *Candida* species, necessitate the understanding of their genetic diversity and epidemiology. MLVA and RAPD-PCR are two molecular typing techniques that have been used to study the genetic characteristics of *C. parapsilosis* and *C. krusei* in clinical samples from animals and humans. Utilizing RAPD assays can enhance *Candida* species identification, augmenting the capabilities of established conventional methods. In this study, we demonstrated the utility of this technique by employing primers OPA2 and NS2 to distinguish between *C. albicans, C. glabrata*, and *C. parapsilosis* [11].

In the study by Madhavan et al. [19], 15 clinical non-albicans *Candida* samples (*C. krusei, C. parapsilosis,* and *C. glabrata*) were isolated from two large hospitals in Kuala Lumpur and their genetic diversity was evaluated using RAPD-PCR. The results showed genetic similarity of 5.12% to 25% observed among different clinical non-albicans *Candida* strains. In that study [19], primers OPA03, OPA02, and OPA08 were used, and those authors stated that this method can be used as a reliable tool for investigating the genetic diversity of different non-albicans *Candida* strains. In the current study, contrary to the study by Madhavan [19][19], the Simpson's coefficient was found to be 31% for *Candida krusei* and 35% for *C. parapsilosis*, leading to the conclusion that despite its ability to classify the genetic strains of the mentioned fungi, RAPD-PCR is not a powerful and reliable tool for investigating the genetic diversity of different non-albicans *Candida* strains, as it lacks sufficient discriminatory power.

The RAPD profiles generated revealed minimal distinctions among strains of the same species, underscoring the challenge of identifying primers capable of detecting intraspecific polymorphisms within these clonal species [20]. The study by Wojciechowska-Koszko [21] investigated the genetic diversity and antifungal susceptibility of *C. parapsilosis* isolated from patients in Poland. 

MLVA is a molecular typing method that has been widely used in epidemiological studies to investigate the transmission dynamics and evolution of various microbial pathogens, including bacteria and fungi [22]. RAPD-PCR, on the other hand, is a PCR-based technique that generates DNA fingerprints by amplifying random regions of the genome [23]. The use of MLVA in this study enabled the identification of 23 different genotypes among the 100 *C. parapsilosis* and *C. krusei* isolates analyzed. This level of genetic diversity is consistent with previous studies that have employed MLVA to investigate the genetic diversity of *Candida* species [24]. For example, Tavanti et al. [25] used MLVA to investigate the genetic diversity of *C. orthopsilosis* clinical isolates and identified 14 different genotypes among 43 isolates. Similarly, Arastehfar et al. [26] used a 9-plex PCR to investigate the genetic diversity of *C. parapsilosis* and identified 24 different genotypes among 88 isolates. MLVA has demonstrated superior discriminatory power compared to RAPD-PCR in distinguishing closely related isolates of *C*. *parapsilosis* and *C. krusei*. The high resolution of MLVA makes it possible to identify subtle genetic variations, facilitating the differentiation of strains within the same clade.

MLVA has demonstrated superior discriminatory power compared to RAPD-PCR in distinguishing closely related isolates of *C. parapsilosis* and *C. krusei* [11]. This is due to several factors that make MLVA a more robust and reliable method for molecular typing and genetic analysis. MLVA uses specific VNTR (Variable Number Tandem Repeat) loci that are highly variable and have a high degree of allelic diversity, which makes it more discriminatory than RAPD-PCR [26]. In contrast, RAPD-PCR uses random primers that may not amplify specific regions of the genome, leading to less consistent and reproducible results. MLVA has been shown to have a higher index of discrimination than RAPD-PCR, which is an important factor in distinguishing closely related isolates [27]. For example, a study comparing MLVA and RAPD-PCR for typing *C. krusei* isolates found that MLVA had an index of discrimination of 0.959, while RAPD-PCR had an index of discrimination of 0.604 [28]. This difference in discriminatory power is due to the use of specific VNTR loci in MLVA, which are more likely to amplify variable regions of the genome than the random primers used in RAPD-PCR. MLVA has been shown to be more reproducible and consistent than RAPD-PCR, which is important for accurate molecular typing and genetic analysis. In a study comparing MLVA and RAPD-PCR for typing *C. parapsilosis* isolates [29], MLVA was found to be more reproducible and consistent than RAPD-PCR, with a higher degree of congruence between different laboratories. This is likely due to the use of specific VNTR loci in MLVA, which are more likely to amplify consistent and reproducible regions of the genome than the random primers used in RAPD-PCR.

The use of RAPD-PCR in our study allowed the identification of 17 different genotypes among the 100 *C. parapsilosis* and *C. krusei* isolates analyzed. This level of genetic diversity is also consistent with previous studies that have employed RAPD-PCR to investigate the genetic diversity of *Candida* species [30], [31]. For example, Kathuria et al. [32] used RAPD-PCR to investigate the genetic diversity of Aspergillus terreus and identified 16 different genotypes among 30 isolates. Similarly, Arendrup et al. [33] used RAPD-PCR to investigate the genetic diversity of *C. krusei* and identified 15 different genotypes among 40 isolates. 

Out findings are consistent with previous studies that have employed MLVA and RAPD-PCR to investigate the genetic diversity of *Candida* species. However, the study by Khodavaisy et al. [34] provides a more comprehensive analysis of the genetic diversity and antifungal susceptibility of *C. parapsilosis* and *C. krusei* isolated from patients in Iran. The study also highlights the importance of using molecular typing and genetic analysis to understand the epidemiology and pathogenesis of these opportunistic fungal pathogens.

In the study by Valerio et al. [35], a total of 13 clinical *Candida* samples were collected and examined using RAPD-PCR. Although 15 primers were used in this study, RAPD-PCR did not have sufficient discriminatory power to separate *C. albicans* and non-albicans strains, and it could only distinguish albicans from non-albicans strains. Therefore, it is not recommended for identifying differences between non-albicans *Candida* strains such as *C. parapsilosis* and *C. glabrata* [35]. The result obtained in the study by Maya Valerio [35] is consistent with the present study, because the low discriminatory power of RAPD-PCR in separating different non-albicans *Candida* strains (such as *C. krusei* and *C. parapsilosis*) was clearly observed.

In the study by Badali et al. [29], the genotyping of clinical isolates of *C. parapsilosis* in Iran was conducted using microsatellite markers. The typing of clinical isolates of *C. parapsilosis* based on microsatellite markers revealed 68 distinct genotypes, among which 57 genotypes were observed once and the remaining 11 were identified multiple times. In the present study, the examination of clinical and animal strains was conducted simultaneously, and as a result, the obtained results are not separate. However, similar to the study by Badali et al. [29], the effectiveness of the microsatellite method for investigating the genetic diversity of *C. parapsilosis* strains was confirmed.

Finally, the study by Khodavaisy et al. [34] provides valuable insights into the genetic diversity and antifungal susceptibility of *C. parapsilosis* and *C. krusei* isolated from patients in Iran. The use of MLVA and RAPD-PCR techniques enabled a more detailed analysis of these isolates, providing important information for the development of effective prevention and treatment strategies. The findings of our study are consistent with previous studies that have employed MLVA and RAPD-PCR to investigate the genetic diversity of *Candida* species, highlighting the importance of using molecular typing and genetic analysis to understand the epidemiology and pathogenesis of these opportunistic fungal pathogens. By analyzing the genetic profiles of C. parapsilosis and *C. krusei* isolates from animal and human samples, MLVA and RAPD-PCR have provided valuable insights into the epidemiology of these opportunistic pathogens. MLVA reveals distinct clusters associated with specific hosts or geographical locations, highlighting potential sources of infection and transmission routes.

The clinical significance of a singular technique, such as RAPD analysis, in determining both species and biotype is most pronounced in the realm of molecular epidemiology. Given the rise in nosocomial infections attributed to *Candida* species, there is an urgent call for a rapid and straightforward procedure enabling analysis of outbreaks and tracking person-to-person transmission associated with these organisms [34]. Consequently, more comprehensive subsequent epidemiological analyses are imperative to ascertain whether genetic similarities merely exist in a characteristic cloned population among these isolates or if hospital procedures have facilitated the dissemination of these agents among patients through cross-infections. Understanding the genetic diversity of *C. parapsilosis* and *C. krusei* is crucial for clinical management, especially concerning antifungal resistance and treatment outcomes. MLVA and RAPD-PCR aid in identifying high-risk strains, tracking nosocomial outbreaks, and guiding infection control measures in healthcare settings [17], [36].

The MLVA technique, which relies on analyzing fragment sizes of four microsatellite markers (Cg4, Cg5, Cg6, and Cg10), is straightforward, discriminatory, and highly reproducible. This method shows promise epidemiologically in distinguishing closely related isolates, making it particularly valuable for investigating nosocomial cross-transmission and the progression from colonization to infection. Integration of additional microsatellite markers, as proposed in other studies [12], [37], may further enhance the discriminatory capacity of our MLVA primer set. Although both MLVA and RAPD-PCR are valuable for molecular typing of *Candida* species, MLVA offers higher discriminatory power and reproducibility compared to RAPD-PCR. MLVA is particularly useful for studying genetic relatedness at a finer scale, while RAPD-PCR is more suitable for rapid screening of large sample sets.

The use of MLVA and RAPD-PCR in molecular epidemiology and genetic analysis has provided valuable insights into the genetic diversity and epidemiology of *C. parapsilosis* and C. krusei, with potential implications for clinical management and infection control strategies. The integration of these techniques with whole-genome sequencing could further enhance our understanding of these opportuniustic pathogens, ultimately leading to more effective and targeted interventions. Molecular typing techniques such as MLVA and RAPD-PCR have transformed the study of genetic diversity and epidemiology of *C. parapsilosis* and *C. krusei*. These methods offer valuable insights into transmission dynamics, antifungal resistance, and clinical management of *Candida* infections, paving the way for more targeted and effective control strategies in animal and human healthcare settings [11].

The results obtained demonstrated that clinical and animal isolates of *C. krusei* and *C. parapsilosis *exhibited a high level of genetic diversity, which was effectively examined by the microsatellite method. This method was capable of distinguishing between strains. Therefore, it can be concluded that the microsatellite genotyping method can be useful for screening during disease prevalence studies, especially in cases involving *C. krusei* and *C. parapsilosis*. However, as the findings indicated, the RAPD-PCR method did not possess sufficient discriminatory power in examining the genetic diversity of *C. krusei* and *C. parapsilosis* strains, and therefore, it is not a reliable method in this context and is not recommended [11], [38].

Through the application of MLVA and RAPD-PCR, researchers have gained insights into the genetic relatedness between human and animal isolates of *C. krusei* and *C. parapsilosis*. These molecular methods have provided valuable information about the epidemiology and transmission dynamics of these fungal species, shedding light on potential sources of infection and transmission routes between humans and animals. Studies have provided insights into the genetic relatedness between human and animal isolates of *C. krusei* and *C. parapsilosis* using MLVA and RAPD-PCR [38], indicating that animals could potentially act as reservoirs for human *Candida* infections. The genetic separation observed between human and animal isolates suggests a possible role of animals in transmitting strains causing human disease, posing a risk to immunocompromised individuals. The application of MLVA and RAPD-PCR techniques has enhanced our understanding of the genetic relationships between human and animal isolates of *C. krusei* and *C. parapsilosis*, contributing significantly to the field of molecular epidemiology and fungal pathogen research. Based on the evidence suggesting that the RAPD-PCR method is more effective than the MLVA method in generating distinct clusters among *Candida krusei* and *Candida parapsilosis* isolates from human and animal sources, the RAPD-PCR technique is recommended for typing these samples, not only in Iran, but also in other regions where similar findings have been observed [38], [39]. Furthermore, it is advised to reevaluate this method with samples from each country worldwide, to enhance the understanding of the genetic relationships between human and animal isolates of *C. krusei* and *C. parapsilosis* on a global scale. The enhanced discriminatory power of RAPD-PCR in distinguishing clusters of these *Candida* species makes it a valuable tool for genetic typing and epidemiological studies, potentially aiding in the identification of sources of infection and routes of transmission in different regions [40] . Therefore, the widespread adoption of RAPD-PCR for typing *C. krusei* and *C. parapsilosis* isolates can contribute significantly to the field of molecular epidemiology and fungal pathogen research, both in Iran and globally [35].

## Conclusions

The molecular typing and genetic analysis of these *Candida* species from patients and animals in Iran have provided valuable insights into the epidemiology and pathogenesis of these opportunistic fungal pathogens. The study has shed light on genetic diversity and antifungal resistance profiles of *C. parapsilosis* and *C. krusei*, offering significant implications for clinical management and surveillance of candidiasis in the region.

### Study limitations

The limited data of *C. krusei* genotypes from different countries prevented the identification of accurate evolutionary routes of commensal and pathogenic strains. Further expansion of the MLST database may promote a better understanding of the mixed evolutionary history of this species.

## Notes

### Competing interests

The authors declare that they have no competing interests.

### Authors’ ORCIDs

- Dorostkar AR: https://orcid.org/0009-0009-9998-9368
- Bayat M: https://orcid.org/0000-0001-8329-4283
- Amini K: https://orcid.org/0000-0002-6419-3417
- Yadegari MH: https://orcid.org/0000-0001-7976-3841

### Funding

None.

## Figures and Tables

**Table 1:**
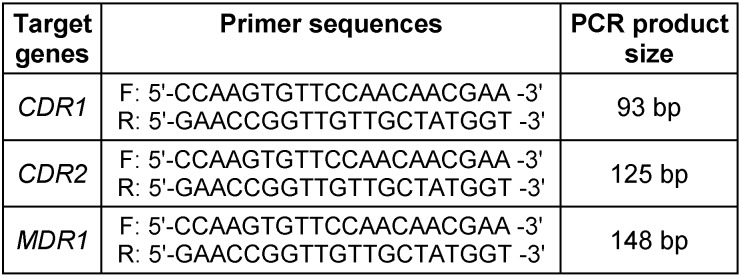
The primers used for antifungal resistance identification

**Table 2:**
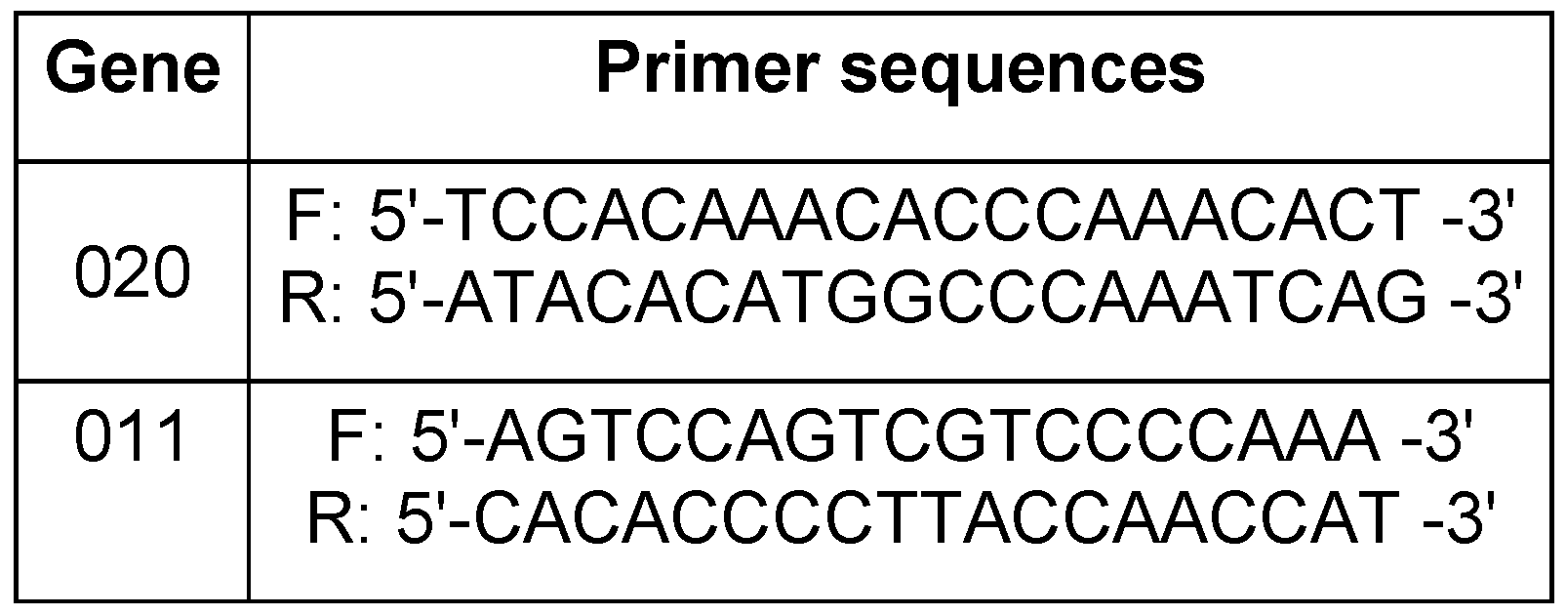
The primers used in the MLVA technique

**Table 3:**
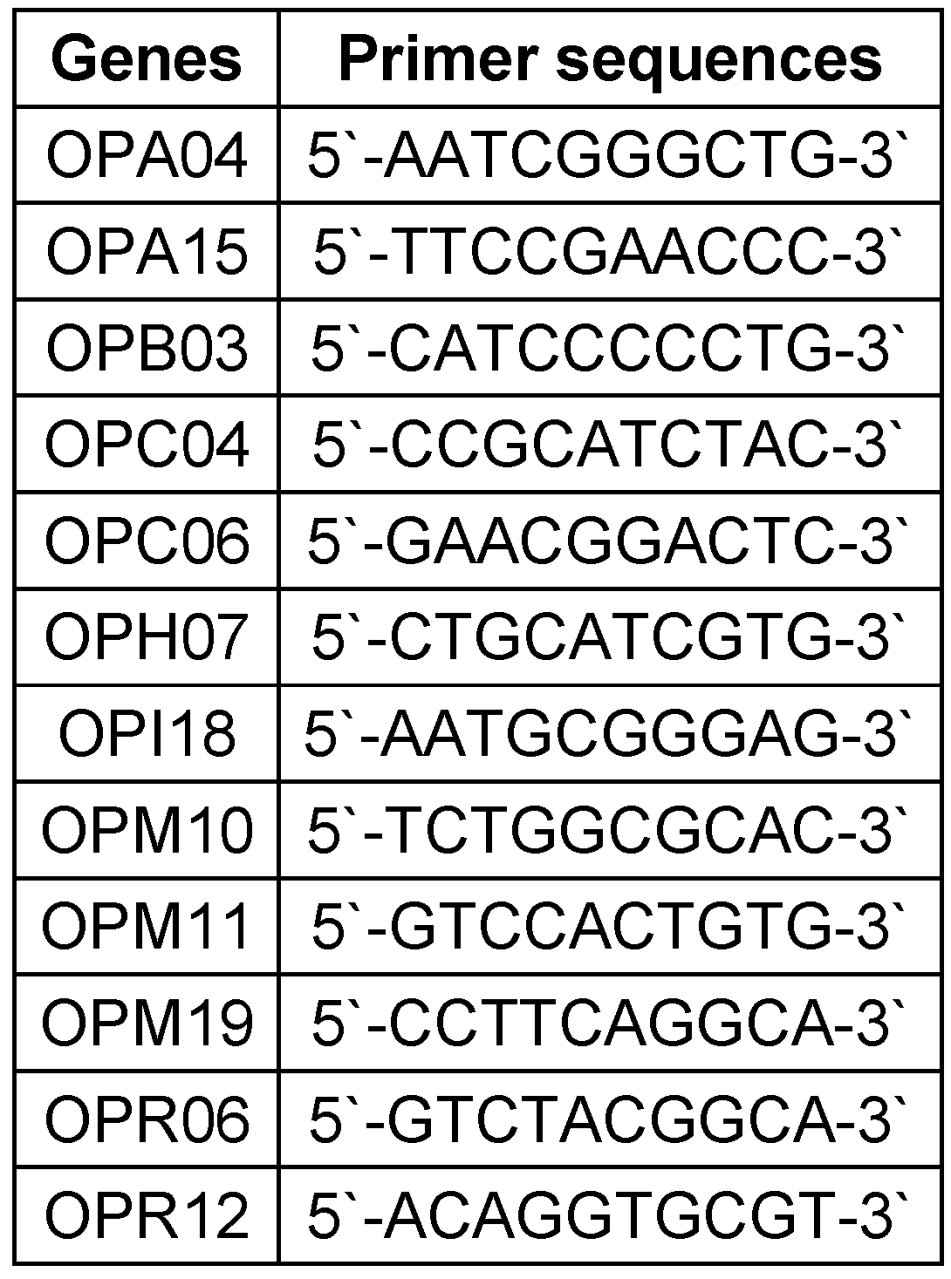
The primers used in the MLVA technique
